# The Color Red Is Implicitly Associated With Social Status in the United Kingdom and China

**DOI:** 10.3389/fpsyg.2018.01902

**Published:** 2018-10-05

**Authors:** Yin Wu, Jingyi Lu, Eric van Dijk, Hong Li, Simone Schnall

**Affiliations:** ^1^Research Center for Brain Function and Psychological Science, Shenzhen University, Shenzhen, China; ^2^Shenzhen Key Laboratory of Affective and Social Cognitive Science, Shenzhen University, Shenzhen, China; ^3^Department of Psychology, University of Cambridge, Cambridge, United Kingdom; ^4^School of Psychology and Cognitive Science, East China Normal University, Shanghai, China; ^5^Department of Social and Organizational Psychology and Leiden Institute for Brain and Cognition, Leiden University, Leiden, Netherlands; ^6^Center for Language and Brain, Shenzhen Institute of Neuroscience, Shenzhen, China

**Keywords:** red, status, color, Implicit Association Test, culture

## Abstract

Research and theorizing on human societies have shown that the color red plays a large role in human psychological functioning. The aim of the present study was to test the association between red and high-status symbols across cultural contexts. Using the Implicit Association Test (IAT) paradigm, across seven experiments (*N* = 357), we demonstrated that participants exhibited a faster association of red color and logos of high-status stimuli compared to red color and logos of low-status stimuli. The effect was shown among both males and females, with two different types of status symbols (car logos and university logos), and with four different contrast colors (white, gray, green, blue). Moreover, this association was observed in both United Kingdom and China. These findings provide compelling evidence for the implicit association between the color red and high social status across two different cultural contexts.

## Introduction

Every object has color. Although colors are objectively specified by lightness, chroma, and hue, they can also carry subjective meaning, especially in social contexts. Indeed, an extensive literature has documented the signal function of the color red, both in humans (Elliot and Maier, 2014) and in animals (Setchell, 2015). For example, in male sticklebacks red coloration has been shown to reflect good health, and therefore females are more likely to choose them as mating partners (Milinski and Bakker, 1990). Similarly, in men red skin, particularly, of the face, reflects high blood oxygenation levels and therefore efficient blood circulation, a feature that is considered attractive by potential romantic partners (Thorstenson et al., 2017). Indeed, some researchers go as far as to suggest that color vision in primates evolved to identify temporary emotional states reflected in skin color (Changizi et al., 2006), for example, to help interpret red skin as an indication of health, attractiveness, or sexual arousal (Drummond and Quah, 2001). Thus, red coloration can signal a number of underlying physical characteristics relevant for social interactions.

A key construct within social life is status, which is defined as an individual’s relative position within their community’s hierarchy in terms of wealth, ability, education, or professional prestige (Kraus et al., 2011). Social status influences both physical health (Sapolsky, 2005) and intellectual achievement (Bate et al., 2013), possibly because high-status individuals have easier access to resources including food, land, and potential mating opportunities; they also have more influence over individuals with lower status (Magee and Galinsky, 2008). Social status can be inferred from various non-verbal cues, such as facial expression (Chiao et al., 2008), body posture (Marsh et al., 2009) and performance hierarchy (Zink et al., 2008).

An important symbolic cue relating to social status is the color red. For example, in mandrill colonies, red color of the face and other parts of the body, including the genitals, is seen by conspecifics to indicate high status, and it therefore elicits submissive behavior in males of a lower social rank (Setchell and Jean Wickings, 2005). Furthermore, when males rise in rank they exhibit an increase in red coloration (Setchell and Dixson, 2001). Beyond signaling biologically based superior fitness and status, however, it has been suggested that in humans certain colors have become symbolically imbued with psychological meaning by way of repeated exposure of certain colors in the context of relevant social situations (Elliot and Maier, 2012).

In human society, there is long tradition that individuals use red to signal power, wealth and status at various situations (Little and Hill, 2007). For example, in ancient societies, it is common that red is used in body decoration and worn on jewelries to represent high rank in ceremonies and rituals (Pickenpaugh, 1997; Orchardson-Mazrui, 1998). Elliot et al. (2010) showed experimentally that men presented against a red background, or wearing a red shirt were perceived as more attractive by women, and this effect was mediated by an increase in social status. Similarly, men consider women to be more attractive and sexually desirable when seen in a visual context that is red (Elliot and Niesta, 2008; Pazda et al., 2012; Schwarz and Singer, 2013; but see Hesslinger et al., 2015; Peperkoorn et al., 2016). In addition, across a range of combat sports in the Olympic Games and in soccer tournaments, red uniforms were shown to be associated with an increased chance of winning the competition (Hill and Barton, 2005).

As a related construct, dominance has been shown to be related to the color red (Mentzel et al., 2017): When participants were asked to indicate whether words printed in different colors described dominance, they were faster and more accurate when classifying dominance words printed in red rather than blue or gray. However, Elliot et al. (2010) emphasize that it is important to distinguish between dominance and status, with the former having a potentially negative meaning that may include being forceful or even aggressive, whereas the latter having a more uniformly positive meaning. We therefore examined the connection between high status and the color red, using positively evaluated logos of high status.

The current research tested whether the color red is also linked to high social status objects which are merely symbolic rather than attached to a specific person in a context of interpersonal attraction or competition. More specifically, we examined symbolic representations of high-status consumer goods and high-status institutions. We investigated the association between red and such social status symbols by using the Implicit Association Test (IAT) paradigm, which assesses implicit social evaluation and has been used extensively in the study of attitudes and stereotype (Greenwald et al., 1998). Using a similar implicit measure, red has been seen to be associated with anger (Fetterman et al., 2011) and danger (Pravossoudovitch et al., 2014), thus suggesting that the IAT method is a useful tool to establish color associations.

Importantly, however, the context can shape the applicability of psychological effects of the color red (Elliot and Maier, 2012; Elliot, 2015). Indeed, red in the context of a physically attractive mating partner can increase liking (Elliot and Niesta, 2008), whereas red in an achievement context (e.g., IQ test) impairs performance because it induces avoidance motivation (Elliot et al., 2007). Thus, while the color red carries significant meaning and has a substantial effect upon individuals’ affect, cognition and behavior, the specific effects can be moderated by the context in which the color appears. One such contextual factor is culture, and an important question for cognitive processes is the extent to which they are culturally universal (Norenzayan and Heine, 2005).

In general, substantial differences have been identified regarding the role of the individualistic vs. collective cultures. More specifically, individualistic cultures (e.g., North America and Europe) value independence based on personal goals and competition (Triandis, 1995). In contrast, collectivistic cultures (e.g., Japan and China) value interdependence, with an emphasis on duties with respect to one’s social community, and deference to those in positions of authority. Resulting differences in self-concept (Markus and Kitayama, 1991) therefore may also be important regarding how people relate to others as part of social hierarchies. Such differences have been found to be reflected in color choices. For example, when students were given colored pencils to fill in geometric patterns, Japanese participants created colorings that were judged to be more harmonious than those of European American participants, whereas the latter created colorings that were seen as more unique (Ishii et al., 2014).

With particular relevance to the current investigation, previous research examined implicit associations between colors and verticality, and found that Mainland Chinese participants tended to associate red with up, and green with down (Jiang et al., 2014). This finding is consistent with earlier work showing that powerful people are represented as being high along the vertical axis, while powerless people are represented as being low (Schubert, 2005). However, the reverse pattern was shown for participants in Hong Kong, which was interpreted to be a consequence of greater exposure of green-high and red-low associations (Jiang et al., 2014). In contrast, the same effects of red color on attraction have been observed both in Western and culturally isolated small-scale societies (Elliot et al., 2013). Thus, some psychological functions of color appear to be based on learning histories whereas others appear to have a more universal basis.

In the present study, we therefore also tested if the red-status association is shared across two distinctive cultures, namely United Kingdom (Experiments 1 and 2) and China (Experiments 3–7), because the United Kingdom is representative of Western culture that values individualism, while China is representative of Eastern culture that values collectivism (Markus and Kitayama, 1991). Indeed, it is possible that only Western cultures, with their emphasis on people’s independent achievement and rank, would show an association between the color red and status. On the other hand, it is also possible that closely knit social communities typical of collectivistic cultures are especially attuned to each person’s relative standing within society.

In the current version of the IAT, participants categorized red color stimuli and logos of high-status cars (or universities) with one key, and contrast color stimuli and logos of low-status cars (or universities) with another key. In a different task block, the pairings were reversed such that red color and logos of low-status cars (or universities) were categorized together while contrast colors and logos of high-status cars (or universities) were categorized together. If participants hold a strong association of red color and high social status, they should respond faster in the first block compared to the second block. Given that previous research observed gender difference in the red effect (Elliot and Maier, 2014), we further tested if males and females showed the association of red and social status to the same extent.

## General Methods

All experiments used the same IAT paradigm. It followed the procedure designed by Greenwald et al. (1998), involving two target categories (high-status vs. low-status stimuli logo) and two color categories (red shape vs. shape of contrast color). Target categories included high- and low-social status stimuli described below. Color categories included different shapes (i.e., triangle, square, hexagon, oval, rectangle) of red or contrast color, five in each category. We used black as background color when presenting the colored shapes on the screen throughout the seven experiments.

We used the RGB (red, green, and blue) color model to produce the color of shapes. The specific parameters were as follows: red (255, 0, 0), white (255, 255, 255), gray (128, 128, 128), blue (0, 0, 255), and green (0, 128, 0). To avoid the influence of authentic color on the high- and low-social status stimuli, we always presented them in gray.

The IAT consisted of seven classification blocks (Greenwald et al., 1998). In the color discrimination task (Block 1, 20 trials), participants were asked to press a left key when a red shape appeared on the screen and a right key for a shape of the contrast color. Similarly, in the initial target-category discrimination task (Block 2, 20 trials), participants discriminated between high-status stimuli (with the left key) and low-status stimuli (with the right key). In the initial association task (Block 3, 20 practice trials; Block 4, 40 data collection trials), the color and target discrimination requirements were combined, so that participants pressed the left key when either a red shape or a high-status logo was presented, and the right key when a shape of contrast color or a low-status logo was presented. In the reversed target-stimuli discrimination task (Block 5, 20 trials), Block 2 was repeated with a switch of the categorization keys (i.e., left key for a low-status logo and right key for a high-status logo appeared.

The reversed association task (Block 6, 20 trials practice; Block 7, 40 trials data collection) again combined two requirements, pressing the left key when either a red shape or a low-status logo was presented and pressing the right key when a shape of white color or a high-status logo was presented. Each block started with brief instructions and a request to respond as fast as possible while trying to minimize errors. Participants were reminded that error rates and response times would be recorded. The trial sequence was randomized for each participant. Half of the participants did the seven blocks in the order presented; to control for order, Blocks 2–4 were swapped with Blocks 5–7 for the other half of the participants.

Participants were 357 university students in both the United Kingdom and China. Using G^∗^Power 3.1 (Faul et al., 2007), we determined that a sample of least 44 individuals for each experiment to have power of 0.90 to detect a medium-size effect (Cohen’s *d* = 0.5). Three participants with an error rate above 30% in the IAT were excluded, following Greenwald et al. (1998). Color-blind individuals were excluded from participation. The study was carried out in accordance with Declaration of Helsinki and was approved by the University of Cambridge Psychology Research Ethics Committee. We report all measures, manipulations, and data exclusions.

## Experiment 1

We first examined the association of the color red and social status with white as the contrast color. We used white since it is the most unobtrusive of the achromatic (i.e., neutral) color in this context (Elliot and Niesta, 2008; Elliot et al., 2010). Logos of high- vs. low- status cars were selected, since cars are ubiquitous in everyday life and unequivocally represent the social economic status of the owners (Dunn and Searle, 2010).

### Method

#### Participants

Sixty university students (30 men, mean age = 22.12 years, *SD* = 2.48, age range = 19–29) at the University of Cambridge, United Kingdom, participated. We recruited Caucasian participants exclusively.

#### Materials and Procedure

We validated the experimental stimuli in an independent United Kingdom student sample (*N* = 89; 34 men). These participants rated the prestige associated with the car logos (1 = lowest, 9 = highest). In line with prediction, high-status cars (i.e., BMW, Ferrari, Maserati, Porsche, Mercedes-Benz; *M* = 7.97, *SD* = 0.70) were rated as more prestigious than low-status cars (i.e., Fiat, Hyundai, Kia, Suzuki, Vauxhall; *M* = 3.32, *SD* = 1.03), *t*(88) = 33.87, *p* < 0.001, Cohen’s *d* = 3.65.

The IAT data were analyzed following the algorithm suggested by Greenwald et al. (1998). The first two trials of either block were excluded due to typically long response latencies. Next, we recoded latencies below 300 ms to 300 ms and those above 3,000 ms to 3,000 ms so that we could eliminate outliers due to inattention or other unusual responses. Data analysis was based on log-transformed response latencies. The IAT effect was calculated as the difference between response times for incompatible trials (high-status stimuli + shape of contrast color, low-status stimuli + red shape) and compatible trials (high-status stimuli + red shape, low-status stimuli + shape of contrast color). Thus, higher scores indicate stronger association between high-social status and red coloration. We used the same statistical method in all subsequent experiments.

### Results and Discussion

Two participants were excluded due to excessive error rate (>30%). The average error rate of the remaining participants was 6.64% (*SD* = 4.37%). As hypothesized, participants responded faster to the high-status car + red shape (*M* = 576 ms, *SD* = 119 ms) and low-status car + white shape than high-status car + white shape and low-status car + red shape (*M* = 667 ms, *SD* = 163 ms). Using log-transformed response time data, a paired *t*-test showed this difference was statistically significant, *t*(57) = −5.49, *p* < 0.001, Cohen’s *d* = 0.72, 95% *CI* = [−0.18, −0.08]^1^. For each participant, we calculated the difference of log-transformed response time data between compatible and incompatible blocks, and tested the gender difference of this red-status association. An independent *t*-test revealed there was no significant gender difference, *t*(56) = 1.51, *p* = 0.137. Thus, this experiment showed that high-status car logos were more easily associated with the color red compared to white, an effect that held for both men and women.

## Experiment 2

Experiment 2 aimed to corroborate the findings in Experiment 1 by using gray as contrast color. Gray is achromatic color, and can vary considerably in lightness, which is different from white (i.e., inherently high in lightness). Thus, red and gray may be equated on lightness, enabling us to test whether these findings are due to lightness differences rather than hue differences.

### Method

#### Participants

Forty-seven university students (21 men, mean age = 24.32 years, *SD* = 3.84, age range = 20–37) at the University of Cambridge, United Kingdom, participated in the experiment. All participants were Caucasian.

#### Materials and Procedure

We used the same set of car logos of Experiment 1.

### Results and Discussion

No participants were excluded due to excessive error rate. The average error rate was 7.55% (*SD* = 4.24%). As hypothesized, participants responded faster to the high-status car + red shape (*M* = 557 ms, *SD* = 99 ms) and low-status car + gray shape than high-status car + gray shape and low-status car + red shape (*M* = 640 ms, *SD* = 156 ms). Using log-transformed response time data, a paired *t-*test showed this difference was statistically significant, *t*(46) = −4.41, *p* < 0.001, Cohen’s *d* = 0.64, 95% *CI* = [−0.16, −0.06]. An independent *t*-test revealed there was no significant gender difference on the red-status association, *t*(45) = 0.06, *p* = 0.956. The experiment therefore replicated the earlier findings with gray as a contrast color.

## Experiment 3

Experiments 1 and 2 showed the association between color red and high-status cars among British participants. The remaining experiments were conducted in mainland of China to test whether cultural context moderated the effect. Experiment 3 first tested if the association between red and high social status generalizes beyond United Kingdom samples. We used a different set of car stimuli that we validated among Chinese participants. Gray was again used as the contrast color.

### Method

#### Participants

Fifty university students (25 men, mean age = 22.64 years, *SD* = 1.79, age range = 20–29) at Shenzhen University, China, participated. All participants were Chinese.

#### Materials and Procedure

Similar to Experiment 1, we validated the experimental stimuli in an independent Chinese sample (*N* = 41; 22 men). These participants rated the prestige associated with a series of cars that are popular in China (1 = lowest, 9 = highest). Again, high-status cars (i.e., BMW, Ferrari, Maserati, Porsche, Mercedes-Benz; *M* = 7.47, *SD* = 0.72) were rated as more prestigious than low-status cars (i.e., Cherry, BYD, Dongfeng, Geely, Great Wall; *M* = 3.64, *SD* = 1.33), *t*(40) = 18.78, *p* < 0.001, Cohen’s *d* = 3.17.

### Results and Discussion

No participants were excluded due to excessive error rate. The average error rate was 3.50% (*SD* = 3.92%). As hypothesized, participants responded faster to the higher-status car + red shape (*M* = 701 ms, *SD* = 167 ms) and lower-status car + gray shape than higher-status car + gray shape and lower-status car + red shape (*M* = 835 ms, *SD* = 189 ms). Using log-transformed response time data, a paired *t*-test revealed this difference was statistically significant, *t*(49) = −6.50, *p* < 0.001, Cohen’s *d* = 0.92, 95% *CI* = [−0.21, −0.11]. A single *t*-test showed there was no significant gender difference on the red-status association, *t*(48) = 0.99, *p* = 0.325.

Given that both Experiments 2 and 3 used car stimuli as symbols of status and gray as contrast color, we tested if the red-status association differed between the British and Chinese sample in a 2 (compatible vs. incompatible trials, within-participant factor) × 2 (British vs. Chinese, between-participant factor) mixed ANOVA. The red-status association was corroborated by a significant main effect of trial type, *F*(1,95) = 59.23, *p* < 0.001. More importantly, the two-way interaction between trial type and sample ethnicity was not significant, *F*(1,95) = 1.95, *p* = 0.17, suggesting the strength of red-status association was equal among our British and Chinese samples.

The main effect of sample ethnicity was significant, *F*(1,95) = 49.01, *p* < 0.001, suggesting that our British participants (*M* = 598 ms) showed overall faster response than Chinese participants (*M* = 768 ms). Note that both Experiments 2 and 3 used cars as representation of social status, however, the specific car brands differed in the low-status category (see the “Materials and Procedure”). Thus, the difference in overall response latency may therefore be due to individuals’ familiarity with the car brands within each culture.

## Experiment 4

Experiment 4 sought to corroborate the finding from Experiment 3 by using blue as a conservative contrast because it is the most commonly selected contrast color in the study of red (Elliot and Niesta, 2008). Moreover, blue is a chromatic color because, like red, it can be equated on chroma as well as lightness, thereby affording another highly controlled test that held these two color properties constant.

### Method

#### Participants

Fifty university students (25 men, mean age = 21.72, *SD* = 2.27, age range = 18–26) at Shenzhen University, China, participated. All participants were Chinese.

#### Materials and Procedure

We used the same set of car stimuli as in Experiment 3.

### Results and Discussion

No participants were excluded due to excessive error rate. The average error rate was 3.25% (*SD* = 2.30%). As expected, participants responded faster to the high-status car + red shape (*M* = 724 ms, *SD* = 142 ms) and low-status car + blue shape than high-status car + blue shape and low-status car + red shape (*M* = 772 ms, *SD* = 180 ms). Using log-transformed response time data, a paired *t*-test showed this difference was statistically significant, *t*(49) = −2.29, *p* = 0.026, Cohen’s *d* = 0.32, 95% *CI* = [−0.09, −0.01]. Again, men and women did not differ, *t*(48) = 0.20, *p* = 0.842, thus confirming that effect occurred universally across both genders and both countries.

## Experiment 5

In Experiment 5, we used green as contrast color, since green is generally associated with positive meanings (Naz and Epps, 2004). More importantly, red and green can be matched on chroma as well as lightness, which allows another highly controlled test of the effect of hue while keeping the other two color attributes the same.

### Method

#### Participants

Fifty university students (30 men, mean age = 23.50 years, *SD* = 2.28, age range = 19–29) at Shenzhen University, China, participated. All participants were Chinese.

#### Materials and Procedure

We used the same set of car stimuli as in Experiment 3.

### Results and Discussion

No participants were excluded due to excessive error rate. The average error rate of the remaining participants was 3.22% (*SD* = 2.59%). As hypothesized, participants responded faster to the high-status car + red shape (*M* = 789 ms, *SD* = 177 ms) and low-status car + green shape than high-status car + green shape and low-status car + red shape (*M* = 868 ms, *SD* = 189 ms). Using log-transformed response time data, a paired *t*-test showed this difference was statistically significant, *t*(49) = −3.17, *p* = 0.003, Cohen’s *d* = 0.42, 95% *CI* = [−0.14, −0.03]. A single *t*-test revealed there was no significant gender difference on the red-status association, *t*(48) = 1.04, *p* = 0.305, replicating the findings of the earlier experiments.

## Experiment 6

Experiments 1–5 had shown an association between high status consumer goods (i.e., cars) and the color red. We also wanted to test the generalizability of this effect to other types of socially relevant status. In Experiment 6, we therefore used logos of universities with either high or low status, to test whether affiliation with a prestigious institution showed similar effects. Gray was used as contrast color.

### Method

#### Participants

Fifty university students (27 men, mean age = 22.20 years, *SD* = 1.59, age range = 20–26) at Shenzhen University, China, participated in the experiment. All participants were Chinese.

#### Materials and Procedure

The same group of independent Chinese participants who rated the stimuli for Experiment 3 also rated the prestige associated with different Chinese university logos (1 = lowest, 9 = highest). The high-status university logos (i.e., Tsinghua, Renmin, Jiao Tong, Wuhan, Sun Yat-sen, and Zhejiang; *M* = 7.59, *SD* = 0.63) were rated as more prestigious than low-status university logos (i.e., Beihua, Bohai, Yangtse, Jianghan, Nantong, North; *M* = 3.94, *SD* = 1.30), *t*(40) = 17.54, *p* < 0.001, Cohen’s *d* = 2.97.

### Results and Discussion

No participants were excluded due to excessive error rate. The average error rate of the remaining participants was 4.38% (*SD* = 3.88%). As expected, participants responded faster to the high-status university logo + red shape (*M* = 830 ms, *SD* = 152 ms) and low-status university logo + gray shape than high-status university logo + gray shape and low-status university logo + red shape (*M* = 969 ms, *SD* = 234 ms). Using log-transformed response time data, a paired *t*-test showed this difference was statistically significant, *t*(49) = −6.32, *p* < 0.001, Cohen’s *d* = 0.99, 95% *CI* = [−0.18, −0.10]. An independent *t*-test revealed there was no significant gender difference on the red-status association, *t*(48) = −0.17, *p* = 0.868. Thus, this effect generalized beyond symbols of exclusive consumer goods (i.e., cars) to symbols of prestigious social institutions (i.e., universities) as indicators of high social status.

## Experiment 7

Experiment 7 sought to replicate the finding from Experiment 6 by using blue as contrast color. Similar to Experiment 4, we used blue as contrast color since it is one of the commonly used contrast color and can be equated on both chroma and lightness with red.

### Method

#### Participants

Fifty university students (26 men, mean age = 21.59, *SD* = 1.70, age range = 19–26) in China participated in the experiment. All participants were Chinese.

#### Materials and Procedure

We used the same set of university stimuli as in Experiment 6.

### Results and Discussion

One participant was excluded due to excessive error rate. The average error rate of the remaining participants was 4.52% (*SD* = 4.42%). Our data analysis included 49 participants. As hypothesized, participants responded faster to the high-status university logo + red shape (*M* = 863 ms, *SD* = 172 ms) and low-status university logo + blue shape than high-status university logo + blue shape and low-status university logo + red shape (*M* = 933 ms, *SD* = 225 ms). Using log-transformed response time data, a paired *t*-test showed this difference was statistically significant, *t*(48) = −2.94, *p* = 0.005, Cohen’s *d* = 0.43, 95% *CI* = [−0.11, −0.02]^2^. A single *t*-test revealed there was no significant gender difference of the red-status association, *t*(47) = −0.21, *p* = 0.839. Thus, using blue color as comparison yielded the same effect as gray.

## Meta-Analysis

To summarize, seven experiments showed the consistent pattern that participants responded faster to compatible trials than to incompatible trials. Importantly, there was no significant gender difference. For further verification, we conducted a meta-analysis to test the statistical replication of the experiments. Using Comprehensive Meta Analysis software, we entered the means, standard deviations, and correlation between log-transformed response time for the compatible and incompatible trials, and sample sizes of each experiment. We did a random-effects meta-analysis of the seven experiments (*N* = 357), which suggested a positive effect of the association of red and high social status, with an effect of medium magnitude, *d* = 0.54, 95% *CI* [0.39, 0.68] (see **Table 1**).

**Table 1:** Results of meta-analysis of the seven experiments.

Experiment	Standard difference in means	Standard error	*Z*-Value	*P*-Value
1	0.643	0.129	4.996	0.000
2	0.609	0.150	4.053	0.000
3	0.802	0.142	5.649	0.000
4	0.277	0.123	2.244	0.025
5	0.445	0.148	3.018	0.003
6	0.681	0.120	5.696	0.000
7	0.331	0.116	2.863	0.004
Combined statistics	0.535	0.075	7.110	0.000

## General Discussion

The present series of experiments provides compelling evidence for the association between the color red and social status. This association was observed in two different countries, namely the United Kingdom and China, by using two different types of status symbols (car logos and university logos), and four different contrast colors (both achromatic and chromatic, namely white, gray, green, and blue). This finding is consistent with the research showing that the color red is implicitly associated with words representing dominance (Mentzel et al., 2017). In particular, individuals respond faster and make fewer errors when categorizing dominance words shown in red. The present research adds to the growing literature showing signaling function of the color red such that red is associated with healthiness, intersexual attraction, dominance and aggressiveness (Elliot and Maier, 2014).

These findings are consistent with evidence of an association of red and social status has received attention in the context of intersexual attraction. Specifically, women perceived men to be more attractive and sexually desirable when men were viewed on a red background or in red clothing. Moreover, this effect is mediated through increased status perception (Elliot et al., 2010). The present study extended these findings by using more objective measurements (e.g., response time) rather than self-report. Notably, Elliot et al. (2010) found that the effect was gender-specific, such that women viewed red as a signal of high status in men, but red has no effect on men’s perception of others’ men. Moreover, recent work also suggests that women find men with redder faces to be more attractive (Thorstenson et al., 2017). Here, we showed there was no gender difference for this effect, suggesting both men and women equally hold the association of red and status.

We observed the red-status association in both United Kingdom and China, two representative cultures from the Western and Eastern society, suggesting this association could be a psychological phenomenon with potentially universal aspects. An important question concerns the potential mechanism behind the observed effect. Although we can only speculate, one possibility relates to testosterone-induced vascularization. Testosterone is a steroid hormone produced primarily by the gonads, and has been linked to social status seeking in humans (Eisenegger et al., 2011). As testosterone has been implicated in peripheral vascular processes, it is possible that high social status is also associated with greater redness, and people acquire this association implicitly. Indeed, one study showed competitors who choose red symbols have higher basal testosterone levels (Farrelly et al., 2013). The testosterone-induced vascularization mechanism provides physiological explanation why the observed red-status link is culturally universal. Further studies are needed to directly test the possible role of testosterone, relative to other social or evolutionary mechanisms, in linking red and social status, an effect that we observed to be universal across two highly different cultures. The second possibility is that the repeated pairing of red color and status through languages, taboos, decorations or rituals in modern society would constitute a more likely explanation to account for the pattern in the study using the IAT. Future studies could fruitfully test the cultural–historical association of red and high-status.

Some limitations should be noted. First, the present research tested the red-status association by using an established implicit measure, however, it is unclear if people hold such association in their explicit knowledge. Future studies are needed to examine this question in a more overt and explicit approach, e.g., self-report. Second, we chose our stimuli based on pre-tested status ratings for the brands. Individual differences in their familiarity with the stimuli could confound their response time. Indeed, a comparison between Experiments 2 and 3 showed that British participants responded faster to the car stimuli than the Chinese participants (see Results and Discussion, Experiment 3), possibly due to the fact that participants from developed countries (i.e., United Kingdom) have more exposure and knowledge about the cars than the participants from developing countries (i.e., China). Future studies need to address this issue by equating familiarity and prototypicality between high- and low- status brands. Third, we only tested Chinese and British participants in our experiments. Although it is well recognized that the two cultures differ largely on collectivism vs. individualism values, future research need to investigate more rigorously the red-status association in other cultural contexts, in order to testify the cultural universality claim (Norenzayan and Heine, 2005).

## Conclusion

In conclusion, across seven experiments, we demonstrate the association between color red and high social status in two distinctive cultures, namely United Kingdom and China. This finding indicates at least some cultural universality in color cognition.

## Author Contributions

YW and SS developed the conceptual background and design for the study, and wrote the paper and the other authors provided comments. YW collected and analyzed the data. All authors approved the final version of the manuscript for submission.

## Conflict of Interest Statement

The authors declare that the research was conducted in the absence of any commercial or financial relationships that could be construed as a potential conflict of interest.

## Footnotes

1 Including the two excluded participants did not change the pattern of results, *t*(59) = −5.304, *p* < 0.001, Cohen’s *d* = 0.82, 95% *CI* = [−0.17, −0.08].

2 Including the participant with excessive error rate didn’t change the pattern of results, *t*(49) = −3.05, *p* = 0.004, Cohen’s *d* = 0.44, 95% *CI* = [−0.11, −0.02].
